# Cariprazine Safety in Adolescents and the Elderly: Analyses of Clinical Study Data

**DOI:** 10.3389/fpsyt.2020.00061

**Published:** 2020-03-03

**Authors:** Balázs Szatmári, Ágota Barabássy, Judit Harsányi, István Laszlovszky, Barbara Sebe, Mónika Gál, Kazushi Shiragami, György Németh

**Affiliations:** ^1^ Department of Clinical Research, Gedeon Richter Plc., Budapest, Hungary; ^2^ Department of Medical Affairs, Gedeon Richter Plc, Budapest, Hungary; ^3^ Department of Clinical Research and Development, Mitsubishi Tanabe Pharma Corporation, Tokyo, Japan

**Keywords:** cariprazine, schizophrenia, adolescents, elderly, safety

## Abstract

Schizophrenia is a life-long mental disorder, affecting young adolescents to elderly patients. Antipsychotic treatment is indicated for all patients with schizophrenia, including the very young and old as well. Developmental issues in the young and decline in organ functioning in the elderly could influence reactions to the drug, and require different dosing regimens. The aim of the present article was to examine the safety profile and dosing requirements in adolescent (13 to less than 18) and elderly (65 and above) patients treated with cariprazine. Data from two clinical studies (one pharmacokinetic pediatric study and one phase III clinical trial) on 49 adolescent patients and 17 elderly patients (65 years of age or above) treated with cariprazine was examined. Safety measures included assessment of adverse events (AEs), clinical laboratory values, physical examinations, extrapyramidal symptom (EPS)-, depression-, and suicidality rating scales. Safety parameters were summarized using descriptive statistics. Results indicate that cariprazine was generally safe and well tolerated. Adverse events in the marginal age populations were comparable to the adult population, except for less insomnia in the young and no reports of akathisia in the elderly. Laboratory parameters, vital sign values and EEG parameters were comparable to previously published data in the adult population. In conclusion, cariprazine in the approved adult dose-range of 1.5–6 mg might be a safe treatment option also in adolescent and elderly patients with schizophrenia. Further studies are need to verify these preliminary findings.

## Introduction

Schizophrenia is a life-long highly disabling mental disorder with an estimated median lifetime prevalence between 0.72% and 0.75% (1, 2). Schizophrenia can occur at any age from childhood to late adulthood. Typically the onset of symptoms is in late adolescence or early adulthood, although cognitive impairments and behavioral changes can be recognized at an earlier age (3). While people with schizophrenia have two- to threefold higher mortality, the course of the disease along with the arising comorbidities with age require special attention in elderly patients (1).

Among pharmacologic interventions, antipsychotics are the first-choice treatment option for schizophrenia, however there are only limited data available in the pediatric (aged between 13 and 18) and the elderly population (aged over 65). In both populations cautious approach is needed when treated with antipsychotics due to various risk factors, such as neurodevelopmental and hormonal changes in the pediatric population and the increasing chance of comorbidities and changing patterns of schizophrenia symptoms in the elderly (4, 5). These factors may warrant a systematic review of available data on antipsychotics to identify special dosing recommendations or precautions.

Early onset schizophrenia (EOS) is a severe, frequently disabling, chronic/recurrent condition with a prevalence that approaches 0.5% in those under the age of 18 years (6). However, the prevalence of child onset schizophrenia (COS—schizophrenia prior to the age of 13 years) is much less frequent with estimates of <0.01%.

While in the literature the prevalence of diagnosed EOS is around 0.5%, some reports note that nearly a third of patients with schizophrenia develop their first psychotic symptoms before the age of 19 years (7–9). There are also references with lower figures with a prevalence of 1 per 500–10,000 in mid-adolescence (10). The prevalence of COS is even lower, generally quoted as 1 in 10,000 (3, 5–7).

The NICE clinical guideline on psychosis and schizophrenia in children and young people notes that the prevalence of psychotic disorders (including but not limited to schizophrenia) in children aged between 5 and 18 years has been estimated to be 0.4%, while the figure across all ages and populations in the UK is 0.7% (11). Schizophrenia accounts for 24.5% of all psychiatric admissions in young people aged 10 to 18 years (the overall admission rate is 0.46 per 1,000 for this age range), with an exponential rise across the adolescent years. The rise in incidence increases most from the age of 15 years upwards.

Due to this very low prevalence of schizophrenia in children below 13 years the European Guideline for the Clinical Development of Medicinal Products for Schizophrenia also recognizes that that pediatric antipsychotic drug development should focus on children aged between 13 up to 18 years (12).

Antipsychotic drugs are widely used in the treatment EOS. Nevertheless, the magnitude of efficacy and tolerability data in this population is well below the information available in adults. Based on this scarce amount of data, antipsychotic treatment is efficacious in children and adolescents with schizophrenia, with some specific aspects such as reduced efficacy and a more severe safety profile compared to adults (13–15). Children and young people are more sensitive to potential adverse effects of antipsychotics, including weight gain, metabolic effects and movement disorders (11). These might be partly explained by the ongoing processes in the developing body of adolescent patients (different maturation of neurotransmitter systems, receptor regulations, metabolism, etc.) (16).

Some differences between adult and pediatric subjects with schizophrenia with regard to neuropathology have been observed. Brain development is still ongoing in the late adolescence making this population more vulnerable to psychological diseases, such as schizophrenia. Neuroimaging studies in subjects with COS who were followed from the age of 6 years to their early 20s showed that during adolescence, there was progressive ventricular enlargement and progressive reduction of cortical grey matter that seemed to be an exaggeration of the normal developmental “pruning” of brain connections observed in late adolescence. This grey matter loss was more severe than was seen in adult-onset schizophrenia in later years. The loss in COS in adolescence seemed to slow down and to be more localized to prefrontal and temporal cortices as compared with most adult studies, establishing biological continuity between childhood onset and adult onset forms. Studies in an ultrahigh risk group, in whom imaging was conducted before and after the onset of psychosis (mean age 19 years), showed grey matter loss in those progressing to psychosis compared with those who did not, with maximal changes localized to the prefrontal cortex. The changes continued over the first 2–4 years of the disease. The pattern of grey matter retraction was an amplification of the normal pattern. In conclusion, the structural changes observed are normal maturational changes in brain regions typical for this age group however at an accelerated speed. Children who develop schizophrenia during this period have a disturbance in their normal maturation process both in grey and white matter, not only structurally but in function as well (17).

These altered needs due to ongoing developmental processes in the adolescent population may call for dose adjustments and specific safety monitoring when treating adolescent patients with schizophrenia.

As schizophrenia usually starts in the late adolescence/early adulthood, the incidence of schizophrenia tends to decline with age. Only about 20% of schizophrenia cases start after the age of 40 (18). Although Diagnostic and Statistical Manual of Mental Disorders 5th edition (DSM–5) does not use the term of late-onset schizophrenia, but states that there are cases when late-onset cases can meet the schizophrenia criteria, the International Late-Onset Schizophrenia Group gives a clear consensus definition for both late-onset and very-late-onset schizophrenia by an onset after 40 and 60 years, respectively (19).

Based on the above, schizophrenia in elderly can be divided into two subgroups: patients with an onset of schizophrenia before mid-life and after mid-life, with some confusion around the exact cut-offs across the definitions.

Although elderly patients with schizophrenia will double by 2025, only about 1% of the publications focus on this population (20).

There is one study conducted among elderly people suffering from schizophrenia in the Amsterdam area, the Netherlands. This study found that the one-year prevalence was 0.55% for schizophrenia (0.71% if schizoaffective and delusional disorders were also taken into account), if divided by onset time the prevalence was for early-onset 0.35%, for late-onset 0.14% and for very-late-onset 0.05%. These numbers are comparable to the prevalence data gained from the younger population (21).

Elderly patients usually suffer from multiple diseases, and use more medications (polypharmacy). As a result of aging, these patients already have dysfunctional organ functioning, which might make them more vulnerable to side effects of drugs. There is an increased risk (about 3.5 times when taking psychotropic medication) that elderly patients develop side effects necessitating hospitalization. In case of antipsychotics most of these events are associated with the typical anticholinergic effects, such as Parkinsonism, tardive dyskinesia, orthostatic hypertension, cardiac conduction disturbances, reduced bone density, sedation and cognitive dysfunction. This is because the pharmacodynamic and pharmacokinetic profiles are significantly altered in the elderly, especially in the presence of various age-related comorbidities and pathologies. As such, reduced absorption and distribution is observed that can be attributed to the reduction of total body water by about 10-15% and the decreased serum albumin levels. The elimination and biotransformation of drugs in the liver is also altered/slower among the elderly because of significant liver volume and blood flow decline. Furthermore, it has been found that other factors such as long-term attachment to bed, dehydration, congestive heart failure, and muscle atrophy, also have a significant effect on pharmacokinetics. Polypharmacy is also really common in elderly, which can have major consequences on the pharmacokinetics of the antipsychotics. Non-compliance is really common in patients with schizophrenia; however, there are further factors can increase the risk of medication non-compliance such as visual impairment, weakened motor skills and cognitive problems (22).

These altered needs due to age associated decline in the elderly may call for dose adjustments and specific safety monitoring when treating elderly patients with schizophrenia.

Cariprazine is an orally active, potent dopamine D_3_/D_2_ receptor partial agonist with preferential binding to D_3_ receptors and partial agonism at serotonin 5-hydroxytryptamine (5-HT_1A_) receptors. It has been approved for the treatment of schizophrenia, bipolar mania and bipolar depression in adults by the Food and Drug Administration (FDA) and for the treatment of schizophrenia in adults by the European Medicinal Agency (EMA).

Cariprazine's efficacy in schizophrenia, which served for approval by the regulatory Agencies, was proven in three double-blind, placebo controlled (two of the studies involved an active arm for assay sensitivity as well) 6-week pivotal studies in acute exacerbation of schizophrenia, one double-blind, placebo controlled, long-term maintenance of effect study and a double-blind, risperidone-controlled 26-week study in patients with predominant negative symptoms of schizophrenia. Furthermore, there were two additional open-label 52-week safety studies in patients with schizophrenia. The FDA and EMA approved dose range for the treatment of schizophrenia is 1.5 to 6.0 mg daily (23–29).

In the targeted dose range of 1.5–6 mg/day, the most frequent treatment-emergent adverse events (TEAEs)—defined as occurring in more than 10% of patients—were akathisia (14.6%), insomnia (14.0%), and headache (12.1%) (30).

The incidence of patients with TEAEs associated with hyperlipidemia was approximately 1% across all treatment groups. The incidence of patients with TEAEs associated with hyperglycemia and diabetes mellitus was < 1% with cariprazine and 1% with placebo. There was no clinically significant difference in the mean change from baseline to end or in shifts from normal values at baseline to high values during the study in fasting triglycerides, cholesterol or serum glucose between cariprazine and placebo-treated patients (31, 32).

There were slightly greater mean increases in body weight and BMI in the cariprazine group compared to the placebo group in the controlled short-term schizophrenia studies. In the long term maintenance of effect study there was no clinically relevant difference in change of body weight from baseline to end of treatment (0.3 kg for placebo, 1.1 kg for cariprazine). In the long term active controlled study, mean change from baseline to end in body weight was -0.36 kg for cariprazine and + 0.64 kg for risperidone (28).

During the short-term and long-term studies, cariprazine treatment was not associated with serum prolactin level increases. Thyroid function changes were small and similar to placebo (25, 28, 31, 32).

In conclusion, cariprazine was generally safe and well tolerated, with most common adverse events related to extrapyramidal symptoms typical for antipsychotics. Otherwise, cariprazine did not increase prolactin levels, did not cause significant weight gain and proved to be metabolically silent, suggesting a safety profile that can be beneficial for both the pediatric and the elderly population.

Only a limited number of patients below the age of 18 or above 65 were enrolled into the cariprazine clinical development program. However, data exists of one pediatric study as well as a study including elderly patients with schizophrenia which is summarized in this article. The aim of this article is to review the available safety data in these patient populations. Since data was collected in a clinical trial setting, the data is well-detailed and of good quality. Safety measures were tailored for the schizophrenic patient population, special attention was given to the uniqueness of the pediatric population when designing of the trial, including the applied safety scales. Data are presented in an age-stratified manner as much as possible in order to be able to determine the similarities between the adolescent, adult, and elderly populations. Differences will also be addressed and potential explanations given where possible. Analyses are focused on the safety and tolerability profile as well as on dosing recommendations of cariprazine in these patient populations.

## Methods

### Cariprazine in Adolescent Population

Cariprazine was examined in an open-label, multinational, multicenter phase I clinical study in pediatric patients with schizophrenia between the age of 13 and less than 18 (EudraCT Number: 2016-002327-29). The primary objectives of the study were to assess the pharmacokinetics, safety and tolerability of cariprazine in adolescents and to determine the dosing recommendations for the pediatric population. No efficacy data was collected in this study.

Patients in the study were enrolled into 3 cohorts based on the cariprazine dose they received. Cohort 1 received 1.5 mg/day, Cohort 2 received 3.0 mg/day, and Cohort 3 received 6.0 mg/day of cariprazine. Each cohort was further divided into age groups. Each cohort included patients in each of the following age ranges: 13 to < 15 years (Age-group A), 15 to < 18 years (Age-group B), and 18 to 40 years (Age-group C). Altogether there were nine subgroups (1A, 1B, 1C, 2A, 2B etc.) based on dose and age-range, with the aim to enroll six patients in all subgroups completing the 28 days treatment and suitable for pharmacokinetic (PK) analyses.

#### Dosing

Dosing duration was 28 d, including an up-titration of cariprazine up to 4 d. Patients taking antipsychotic medication had to have their previous drug down-titrated and discontinued by the fifth day of cariprazine dosing. Due to the enrollment of a pediatric population, a very cautious approach was applied for dose escalation: not only by dose strength as the usual approach for such studies but by age groups as well. The dosing of the first patient in the younger pediatric age group was only allowed after the first patient in the older pediatric age group had reached his or her fifth day of dosing in the given dose strength. The second and subsequent patients in the younger age group were only allowed to be enrolled once the first patient had reached the fifth day of dosing without any tolerability concerns or major safety findings.

Cariprazine has two active metabolites: desmethyl-cariprazine (DCAR) and didesmethyl-cariprazine (DDCAR). The functional half-life of cariprazine and the first metabolite (DCAR) is ~2 d, of the second metabolite (DDCAR) 8 d and overall ~1 week for total cariprazine (the sum of all three). The plasma concentration of total cariprazine will gradually decline following the discontinuation of the drug. The plasma concentration of total cariprazine decreases by 50% in ~1 week and greater than 90% decline in total cariprazine concentration occurs in ~3 weeks. Due to this pharmacokinetic profile, after 28 d of dosing, cariprazine was discontinued without down-titration taking special attention for potential withdrawal symptoms during the 14-d follow-up period.

#### Safety Measures

Safety assessments were carefully selected for this patient population. Standard clinical trial safety measurements were included such as spontaneous adverse event reporting, physical examination, clinical laboratory (hematology, serum chemistry, prolactin measurement and urinalysis), electrocardiogram (ECG), and vital signs (blood pressure, pulse rate, body temperature, and body weight) collection. Movement disorders are common side effects of antipsychotics, therefore specific scales were applied: the Barnes Akathisia Rating Scale (BARS), the Abnormal Involuntary Movement Scale (AIMS) and the Simpson Angus Scale (SAS). The definition for treatment-emergent akathisia was if the patient’s BARS score was ≤ 2 at baseline and > 2 at any treatment phase assessment. A patient was considered to have treatment-emergent Parkinsonism if the patient's SAS score was ≤ 2 at baseline and > 2 at any treatment phase assessment. Further measurements evaluating antipsychotic related side effects were the Columbia Suicide Severity Rating Scale (C-SSRS) used for the assessment of suicidality, and the Children's Depression Rating Scale-Revised (CDRS-R) for the evaluation of depressive symptoms.

Side effects can highly affect drug compliance which is crucial in clinical studies primarily aiming to assess the pharmacokinetic parameters of a drug. Moreover, for various reasons pediatric patients might not be able to identify or willing to verbalize adverse events as adults, resulting in underreporting of adverse events. For this reason, additionally to the standard adverse event questioning, a systematic adverse event collection was also done by using the Udvalg for Kliniske Undersøgelser (UKU) side effects rating scale which is a comprehensive rating scale to assess the side effect of psychotropic medications (33).

### Cariprazine in the Elderly Population

Cariprazine was examined in a 48-week open-label trial conducted in Japan enrolling patients with chronic phase of schizophrenia and elderly patients—ages between 65 and 74 years (ClinicalTrials.gov. NCT01625897, study A002-A7). The primary objective of the study was to assess the long-term safety, tolerability, and efficacy of cariprazine in this patient population. The cariprazine schizophrenia development program, as mentioned above, did not include patients under the age of 18 and above the age of 65. Adults above 65 years of age were not included in the studies, due to safety considerations associated with antipsychotic use in the elderly, including increased cardio- and cerebrovascular diseases, increased risk of mortality especially in elderly patients with dementia, increased hepatic and renal impairment; and polypharmacy. However there were three patients (one from a short-term placebo-controlled study and the additional two patients in the predominant negative symptoms study) above 65 years of age, who provide additional data.

#### Dosing

Patients had been randomized to either cariprazine or risperidone and after a fix dose period of 2–4 weeks patients continued up to 48 weeks on the respective treatment arm with a flexible dosing of 1.5–9 mg/day (1.5 mg, 3 mg, 6 mg, or 9 mg) of cariprazine or 2–12 mg/day (2 mg, 4 mg, 6 mg, 8 mg, 10 mg, or 12 mg) of risperidone. However, while risperidone doses were allowed to be changed after two weeks of treatment, for the cariprazine treated patients this was only allowed after week 4 due to the pharmacokinetics of the compound.

#### Safety Measures

Most of the safety measures were consistent with previous studies of the clinical development of cariprazine. Standard clinical trial safety measurements were included such as spontaneous adverse event reporting, clinical laboratory (hematology, serum chemistry, prolactin measurement, and urinalysis), vital signs, and ECG collection. Vital sign assessment included systolic and diastolic blood pressure, pulse rate, body temperature, body weight, waist circumference and BMI. PCS criteria were set for blood pressure, pulse rate, and body weight. Furthermore, ophthalmologic assessment, measurements evaluating antipsychotic related side effects were also applied: BARS, AIMS and the Drug-induced Extrapyramidal Symptoms Scale (DIEPSS) instead of the SAS and C-SSRS for suicidality. A patient was considered to have treatment-emergent Parkinsonism if the patient scored on items 1–5 of the DIEPSS three or higher for at least one item or two or higher for at least two items at any postbaseline time point, or had an increase from baseline of at least 3 points in the total score of items 1–5. The same definition was used for the treatment-emergent akathisia as in the pediatric study (BARS total score was ≤2 at baseline and >2 postbaseline).

## Results

### Cariprazine in the Pediatric Population

#### Patient Disposition and Demographics

Twenty-two (88.0%) patients in Age group A, 21 (87.5%) patients in Age group B, and 20 (90.9%) patients in Age group C had been enrolled into the study and received at least one dose of the investigational medicinal product (IMP): Safety Analysis Population (**Table 1**). One (10.0%) patient in Age group A (Cohort 2) terminated the study early due to an AE of acute tonsillitis and one (11.1%) patient in Age group B (Cohort 2) terminated the study early due to withdrawal of informed consent.

**Table 1:** Patient disposition, Safety Analysis Population (All Screened Patients).

Parameter	Cohort 1 1.5 mg/day n (%)	Cohort 2 3.0 mg/day n (%)	Cohort 3 6.0 mg/day n (%)	Overall n (%)
**Age-group A (13 to < 15 years)**	6	10	6	**22**
Patients Screened	–	–	–	25
Screen Failure	–	–	–	3 (12.0)
**Safety Analysis Population**	**6**	**10**	**6**	**22 (88.0)**
Patients completing the Treatment Phase	6 (100.0)	9 (90.0)	6 (100.0)	21 (84.0)
Patients completing the Follow-up Phase	6 (100.0)	9 (90.0)	6 (100.0)	21 (84.0)
Patients terminating the study early	0	1 (10.0)	0	1 (4.0)
**Age-group B (15 to < 18 years)**	6	9	6	**21**
Patients Screened	–	–	–	24
Screen Failure	–	–	–	3 (12.5)
**Safety Analysis Population**	**6**	**9**	**6**	**21 (87.5)**
Patients completing the Treatment Phase	6 (100.0)	8 (88.9)	6 (100.0)	20 (83.3)
Patients completing the Follow-up Phase	6 (100.0)	8 (88.9)	6 (100.0)	20 (83.3)
Patients terminating the study early	0	1 (11.1)	0	1 (4.2)
**Age-group C (18 to 40 years)**	6	8	6	**20**
Patients Screened	–	–	–	22
Screen Failure	–	–	–	2 (9.1)
**Safety Analysis Population**	**6**	**8**	**6**	**20 (90.9)**
Patients completing the Treatment Phase	6 (100.0)	8 (100.0)	6 (100.0)	20 (90.9)
Patients completing the Follow-up Phase	6 (100.0)	8 (100.0)	6 (100.0)	20 (90.9)
Patients terminating the study early	0	0	0	0

Patients excluded from the PK analyses were replaced to have six patients in each subgroup.

Overall, the demographic characteristics were comparable across Cohorts in each of Age groups A, B, and C (**Table 2**).

**Table 2:** Demographics and other baseline characteristics (Safety Analysis Population).

Parameter	Cohort 1 1.5 mg/day n (%)	Cohort 2 3.0 mg/day n (%)	Cohort 3 6.0 mg/day n (%)	Overall n (%)
**Age-group A (13 to < 15 years)**	**n = 6**	**n = 10**	**n = 6**	**N = 22**
**Age (years) at Informed Consent**				
Mean	13.5	13.4	13.3	13.4
SD	0.55	0.52	0.52	0.50
**Sex**				
Female	1 (16.7)	1 (10.0)	2 (33.3)	4 (18.2)
Male	5 (83.3)	9 (90.0)	4 (66.7)	18 (81.8)
**Race**				
Black or African American	0	0	0	0
Asian	0	0	0	0
White	6 (100.0)	10 (100.0)	6 (100.0)	22 (100.0)
Other	0	0	0	0
Multiple	0	0	0	0
**Height (cm) at Screening**				
Mean	162.5	162.1	155.2	160.3
SD	11.20	9.01	2.32	8.74
**Weight (kg) at Screening**				
Mean	58.80	55.31	52.17	55.40
SD	11.024	7.205	7.839	8.491
**Age-group B (15 to < 18 years)**	**n = 6**	**n = 9**	**n = 6**	**N = 21**
**Age (years) at Informed Consent**				
Mean	16.7	15.8	15.8	16.0
SD	0.52	0.67	0.41	0.67
**Sex**				
Female	4 (66.7)	4 (44.4)	1 (16.7)	9 (42.9)
Male	2 (33.3)	5 (55.6)	5 (83.3)	12 (57.1)
**Race**				
Black or African American	0	0	0	0
Asian	0	0	1 (16.7)	1 (4.8)
White	6 (100.0)	9 (100.0)	5 (83.3)	20 (95.2)
Other	0	0	0	0
Multiple	0	0	0	0
**Height (cm) at Screening**				
Mean	165.7	166.8	169.3	167.2
SD	10.03	8.69	6.74	8.30
**Weight (kg) at Screening**				
Mean	59.80	63.53	64.57	62.76
SD	11.448	8.467	8.376	9.103
**Age-group C (18 to 40 years)**	**n = 6**	**n = 8**	**n = 6**	**N = 20**
**Age (years) at Informed Consent**				
Mean	28.8	27.8	30.8	29.0
SD	8.61	9.00	7.70	8.17
**Sex**				
Female	2 (33.3)	2 (25.0)	2 (33.3)	6 (30.0)
Male	4 (66.7)	6 (75.0)	4 (66.7)	14 (70.0)
**Race**				
Black or African American	0	0	0	0
Asian	0	0	0	0
White	6 (100.0)	8 (100.0)	6 (100.0)	20 (100.0)
Other	0	0	0	0
Multiple	0	0	0	0
**Height (cm) at Screening**				
Mean	170.2	173.1	173.8	172.5
SD	6.59	11.03	12.32	9.93
**Weight (kg) at Screening**				
Mean	73.10	75.26	71.47	73.48
SD	14.201	12.746	12.750	12.584

Patients in Age group A had a mean age of 13.4 years and the majority of patients were male (81.8%). Patients in Age group B had a mean age of 16 years and while overall the majority were male (57.1%), in Cohort 1 the majority of patients were female (66.7%). Patients in Age group C had a mean age of 29 years with male dominance as well (70.0%).

Except for one Asian patient in subgroup 3B all other patients were Caucasian. In order to be eligible in this study, all patients had a medical history of schizophrenia, schizoaffective disorder, or schizophreniform disorder. No patients had clinically significant previous medical and surgical history. In all age-groups psycholeptics (the most common were risperidone, aripiprazole, and olanzapine) were the most frequently reported concomitant medication (between 63.6 and 90.5% depending in the age-group).

#### Safety Measurements

##### Adverse Events and UKU Side Effects Rating Scale

No deaths or serious adverse events were reported in this study. More TEAEs and study drug-related TEAEs were reported for Age group B than were reported for Age groups A and C; the number of patients who reported AEs in Age groups A and C were similar. No clear trend with dose in the number of TEAEs or study drug-related TEAEs was observed (**Table 3**).

**Table 3:** Overall summary of treatment-emergent adverse events (Safety Analysis Population).

	Cohort 1 (1.5 mg/day) (N = 6)		Cohort 2 (3.0 mg/day) (N = 10)		Cohort 3 (6.0 mg/day) (N = 6)		Overall (N = 22)	
	Patients n (%)	Events n	Patients n (%)	Events n	Patients n (%)	Events n	Patients n (%)	Events n
**Age-group A (13 to < 15 years)**
TEAE	2 (33.3)	2	6 (60.0)	15	3 (50.0)	3	11 (50.0)	20
ADR	1 (16.7)	1	2 (20.0)	5	2 (33.3)	2	5 (22.7)	8
SAE	0	0	0	0	0	0	0	0
NEAE	0	0	1 (10.0)	2	1 (16.7)	1	2(9.1)	3
TEAE Leading to Early Termination	0	0	1 (10.0)	4	0	0	1 (4.5)	4
**Age-group B (15 to < 18 years)**	**(N = 6)**		**(N = 9)**		**(N = 6)**		**(N = 21)**	
	**Patients** **n (%)**	**Events** **n**	**Patients** **n (%)**	**Events** **n**	**Patients** **n (%)**	**Events** **n**	**Patients** **n (%)**	**Events** **n**
TEAE	4 (66.7)	26	2 (22.2)	22	5 (83.3)	18	11 (52.4)	66
ADR	4 (66.7)	25	2 (22.2)	20	4 (66.7)	16	10 (47.6)	61
SAE	0	0	0	0	0	0	0	0
NEAE	0	0	0	0	1 (16.7)	1	1 (4.8)	1
TEAE Leading to Early Termination	0	0	0	0	0	0	0	0
**Age-group C (18 to 40 years)**	**(N = 6)**		**(N = 8)**		**(N = 6)**		**(N = 20)**	
	**Patients** **n (%)**	**Events** **n**	**Patients** **n (%)**	**Events** **n**	**Patients** **n (%)**	**Events** **n**	**Patients** **n (%)**	**Events** **n**
TEAE	2 (33.3)	6	4 (50.0)	6	3 (50.0)	6	9 (45.0)	18
ADR	1 (16.7)	2	3 (37.5)	5	2 (33.3)	5	6 (30.0)	12
SAE	0	0	0	0	0	0	0	0
NEAE	2 (33.3)	3	1 (12.5)	1	0	0	3 (15.0)	4
TEAE Leading to Early Termination	0	0	0	0	0	0	0	0

TEAE, Treatment-emergent Adverse Event; SAE, Serious Adverse Event; NEAE, Newly-emergent Adverse Event; ADR, Adverse Drug Reaction.

Treatment-emergent adverse events (TEAE) are adverse events that were not present before the first dose of the investigational medicinal product or if it was present the severity increased after the first dose of IMP. Newly-emergent adverse events (NEAS) are defined as adverse events occurring during the follow-up period if the AE was not present before the start of the follow-up period or was present before the start of the follow-up period and increased in severity during the follow-up period. Adverse drug reactions (ADR) are adverse events that are rated by the investigator as related to treatment.

In Age-group A the most frequent TEAE (occurring in ≥ 5% of patients in either cohort) was sedation in 3 (13.6%) patients. All TEAEs were of mild or moderate intensity, except for the TEAEs of headache and tonsillitis, which were of severe intensity, reported in one patient in Cohort 2; both of these TEAEs were unrelated to the study drug, according to the judgment of the investigator.

In Age-group B the most frequent TEAEs (occurring in ≥ 5% of patients in either cohort) were akathisia and somnolence in five (23.8%) patients, tension headache in four (19.0%) patients, nausea in three (14.3%) patients, and dizziness postural, sedation, fatigue, abnormal dreams, sleep disorder, and hypotension in two (9.5%) patients. All TEAEs were of mild or moderate intensity, except the TEAE of somnolence, which was of severe intensity, reported in 1 patient in Cohort 3, and which was related to the study drug, according to the judgment of the investigator.

In Age-group C the most frequent TEAEs (occurring in ≥ 5% of patients in either cohort) were asthenia and somnolence in two (10.0%) patients and sinus arrhythmia, tachycardia, constipation, dry mouth, nausea, nasopharyngitis, disturbance in attention, paraesthesia, tremor, sleep disorder, tension, hyperhidrosis, and hypotension in one (5.0%) patient each. All TEAEs were of mild or moderate intensity.

The UKU did not reveal any further significant side effects not already captured by the adverse event collection or cannot be attributed to the underlying condition. In the pediatric age-groups only tension headache, emotional indifference, sleepiness/sedation and concentration difficulties were reported as severe by one patient each.

##### Clinical Laboratory

Mean change from baseline to end in all of the clinical laboratory parameters were small and not clinically meaningful.

##### Vital Signs and ECG

Mean change from baseline to end of treatment for vital signs were small and not clinically meaningful. Overall, there were a higher number of patients with predefined potentially clinically (PCS) significant postbaseline vital sign values in Age group B compared to Age group A and Age group C, however, no trend could be observed in any of the PCS values.

12-lead ECG recordings did not show any clinically meaningful differences between the dose cohorts and the age-groups either. PCS values were measured only in few patients and more frequently in the adults compared to the pediatric age-groups.

##### Treatment-Emergent Parkinsonism and Akathisia

Treatment-emergent Parkinsonism was not captured by the SAS in any of the patients. Treatment-emergent akathisia was only observed in age-group B in all dose levels without showing a dose relationship.

### Cariprazine in the Elderly Population

#### Patient Disposition and Demographics

In study A002-A7 125 patients had been randomized, 83 patients received cariprazine, and 42 risperidone. Out of the 125 patients, 27 were in the elderly age group (≥ 65 years at informed consent) of whom 17 received cariprazine and 10 risperidone (**Table 4**).

**Table 4:** Patient disposition (Safety Analysis Population, A002-A7).

	Total		Elderly	
	Cariprazine (N = 83) n (%)	Risperidone (N = 42) n (%)	Cariprazine (N = 17) n (%)	Risperidone (N = 10) n (%)
**Safety Analysis Population**	**83 (100.0)**	**42 (100.0)**	**17 (100.0)**	**10 (100.0)**
Completed Treatment Period	31 (37.3)	28 (66.7)	8 (47.1)	8 (80.0)
Discontinued Treatment Period	52 (62.7)	14 (33.3)	9 (52.9)	2 (20.0)
Withdrawal of Consent	25 (30.1)	3 (7.1)	2 (11.8)	1 (10.0)
Adverse Event	27 (32.5)	10 (23.8)	7 (41.2)	1 (10.0)
Other	0 (0.0)	1 (2.4)	0 (0.0)	0 (0.0)

In the elderly group, the baseline characteristics were similar between the two treatment groups with some minor differences: there were more females in the cariprazine group than males and the duration of illness was slightly shorter for the cariprazine group than for the risperidone group (**Table 5**). All patients in the elderly population had previously taken antipsychotic medication.

**Table 5:** Demographics and other baseline characteristics (Safety Analysis Population, A002-07).

Demographic Parameter	Total		Elderly	
	Cariprazine (N = 83) n (%)	Risperidone (N = 42) n (%)	Cariprazine (N = 17) n (%)	Risperidone (N = 10) n (%)
**Race, n (%)**				
Japanese	83 (100.0)	41 (97.6)	17 (100.0)	10 (100.0)
Asian (Non-Japanese)	0 (0.0)	1 (2.4)	0 (0.0)	0 (0.0)
Non-Asian	0 (0.0)	0 (0.0)	0 (0.0)	0 (0.0)
**Gender, n (%)**				
Male	39 (47.0)	24 (57.1)	6 (35.3)	5 (50.0)
Female	44 (53.0)	18 (42.9)	11 (64.7)	5 (50.0)
**Age (years)**				
Mean (SD)	47.7 (14.6)	48.9 (13.9)	67.8 (2.5)	66.8 (2.0)
Min, Max	20, 72	27, 70	65, 72	65, 70
**Height (cm)**				
Mean (SD)	162.24 (8.96)	163.04 (9.34)	155.54 (7.67)	158.04 (8.04)
**Weight (kg)**				
Mean (SD)	65.24 (14.48)	64.94 (13.87)	58.64 (11.73)	56.85 (10.97)
**BMI (kg/m^2^)**				
Mean (SD)	24.65 (4.49)	24.38 (4.55)	24.07 (3.56)	22.76 (4.18)
**Duration of Schizophrenia (years)**				
Mean (SD)	19.53 (14.53)	21.50 (15.28)	35.58 (11.59)	39.72 (10.66)

#### Safety Measurements

##### Adverse Events

In the elderly population 16/17 (94.1%) patients in the cariprazine group and 10/10 (100%) in the risperidone group experienced at least one TEAE (**Table 6**). Eleven of the cariprazine treated and nine of the risperidone treated patients had at least one adverse drug reaction, while the number of patients with at least one serious adverse event was four and one, respectively. The number of patients having an adverse event leading to discontinuation was higher in the cariprazine group compared to the risperidone arm. There were no death cases. Due to the limited patient numbers, the most common adverse events were defined as adverse events that had been reported by more than two patients. In the cariprazine arm the most frequent adverse events were schizophrenia, nasopharyngitis, insomnia, hypertension and weight increased. In the risperidone group the most frequently reported TEAEs were hyperprolactinaemia, insomnia and Parkinsonism, which in line with the safety profile of risperidone.

**Table 6:** Overall summary of treatment-emergent adverse events (Safety Analysis Population).

	Elderly	
	Cariprazine (N = 17) n (%)	Risperidone (N = 10) n (%)
Treatment Period and Follow-up Period		
Patients with at least one TEAE	16 (94.1)	10 (100.0)
Patients with at least one NEAE	10 (58.8)	5 (50.0)
Patients with at least one ADR	11 (64.7)	9 (90.0)
Patients with at least one SAE	4 (23.5)	1 (10.0)
Patients who Died	0 (0.0)	0 (0.0)
Patients leading to discontinuation due to TEAEs	7 (41.2)	1 (10.0)

N, Number of patients in Safety Analysis Population; n, Number of patients within each category.

TEAE, Treatment-emergent Adverse Event; NEAE, Newly Emergent Adverse Event; ADR, Adverse Drug Reaction; SAE, Serious Adverse Event.

Patients who were ≥ 65 years at informed consent were defined as Elderly.

##### Clinical Laboratory

The overall changes were small and not clinically significant in the clinical laboratory parameters. The metabolic parameters (including total cholesterol, LDL, HDL and triglycerides) did not show significant changes during the study. The only exception is glucose, which increased in the cariprazine treatment group from a mean baseline of 104.9 mg/dl by 20.8 mg/dl by the end of the study. Prolactin levels increased with risperidone treatment from 23.614 to 62.486 ng/ml, while on the cariprazine group the prolactin levels decreased from 19.325 to 5.429 ng/ml.

The number of PCS laboratory changes during the entire treatment period were generally low. The parameters with PCS values in at least two patients were hemoglobin decrease, hematocrit decrease and LDL increase in the risperidone treatment group and blood glucose increase, creatine kinase increase, glucose in urine and occult blood in urine in the cariprazine treated patients.

##### Vital Signs and ECG

The mean changes from baseline to end were small and not clinically relevant. PCS values measured by at least two patients during the study period were systolic blood pressure decrease, diastolic blood pressure decrease, body weight increase in the risperidone group and diastolic blood pressure increase, body weight decrease in the cariprazine group.

The ECG analyses did not show any PCS values in the elderly population except for one patient with a QTcF value of 503 ms at one study visit in the cariprazine group.

##### Treatment-Emergent Parkinsonism and Akathisia

There were more patients who experienced treatment-emergent Parkinsonism in the risperidone (40%) than in the cariprazine group (17.6%). No treatment-emergent akathisia was measured by the BARS on any of the treatment arms.

Among the three elderly patients from the other phase II/III studies, one patient experienced one TEAE of shortness of breath, that was considered to be related to the study drug.

## Conclusions

Due to the natural course of schizophrenia, spanning from early adolescence to the elderly population, it is important to understand the differences in the safety profile and effectiveness of a given antipsychotic across the different age ranges. Authorities acknowledge this need and request drug manufacturers to develop antipsychotic medication for the adolescent population as part of pediatric investigational commitments. For the elderly, the limited information arising from the standard clinical development programs leads to a dosing recommendation more cautious than for the younger adult population. Authorities do not usually request specific studies, however recommend to collect all data in the elderly population as these are classified missing information.

In the pediatric population, cariprazine was generally well tolerated throughout the 4-week treatment period, without any serious adverse events or early terminations related to the study drug. Generally findings were in line with previous safety findings in the adult population.

There was no clear trend observed that the number of TEAEs would be higher in the younger age group, or that the incidence of TEAEs would increase with the dose. Furthermore, most of the TEAEs reported were mild to moderate of intensity. Akathisia was only captured in the older adolescent population by the predefined treatment-emergent akathisia criterion on the BARS without suggesting a dose relationship to this side effect. No clinically significant differences were observed in any of the clinical laboratory, vital sign, or ECG assessments.

Of note, insomnia did not occur as a reported adverse event; but rather TEAEs of sedation, somnolence or fatigue. Although these TEAEs were also reported in the adult studies, their frequency was significantly lower than that of insomnia. One explanation could be the requirement for patients in the pediatric study to take cariprazine capsules in the morning, while such strict criterion was not set in the adult studies.

In the elderly population the same conclusion can be drawn. Adverse events were generally in line with what has been observed in the adult population previously, with the exception of akathisia, which did not occur in the elderly and was also not captured by BARS. This could be attributed to the limited patient numbers. Another possible reason could be that this patient population is in the chronic phase of schizophrenia with decades of antipsychotic treatment. EPS is the most common side effect of antipsychotics but for which patients can develop tolerability over time. Therefore chronic patients might not be as sensitive to EPS-related side effects of a partial dopamine agonist. In terms of body weight, some weight increase was reported, however PCS weight gain did not occur. There was a slight increase in blood glucose values and reports of diabetes mellitus in two patients. There were two cases of PCS diastolic blood pressure increases during the treatment period but none were present at the end of the study. For ECG results, one cariprazine treated patient had an intermittent elevation of QTcF value.

The main limitation of the presented data—and this is relevant for both age groups—is that efficacy parameters were not assessed since the main goal was to characterize the safety of cariprazine in these patient populations. Further general limitations are that both studies were open-label studies and the number of targeted subjects was low. Therefore, the presented safety data can provide only a signal about the safety profile of cariprazine in these age groups and further studies are needed to verify the results. On the other hand, the presented safety data doesn't reflect different safety profile for cariprazine as it was characterized on a large number of adult subjects during short or long-term treatment.

There are also some study-specific limitations of the presented data. In study A002-A7 only 17 elderly patients—ages between 65 and 74 years—were treated with cariprazine and the selected dose range was 1.5–9 mg/day which is broader than the finally approved 1.5–6 mg/day dose range for cariprazine for the treatment of schizophrenia. Because the dosing design was flexible, treated subjects were not equally distributed to the investigated dose levels of 1.5, 3, 6, and 9 mg/day. The 4.5 mg/day dose was not investigated at all. The presented safety data is related to Japanese patients only which can be also a limiting factor.

In the pediatric study only 6–10 subjects were treated with 1.5, 3, or 6 mg/day cariprazine per age groups (13 to < 15 years and 15 to < 18 years). This patient number is low to judge the possible tolerance difference between the 13 to < 15 years and 15 to < 18 years age groups. The 4.5 mg/day dose was not investigated in the pediatric study either. The duration of the study was 28 days, and this is probably not sufficiently long to assess the long-term safety of cariprazine that can be crucial in case of some safety measures such as metabolic parameters or weight gain. All in all, although there are limiting factors, the presented safety data of the adolescent population did not differ significantly from the adult safety data.

In conclusion, based on the results above the safety profile of cariprazine is similar throughout the age groups from 13 to 74 years. Further specific studies are needed to better understand the long-term safety of cariprazine in the pediatric population and the elderly. So far the data is encouraging also in terms of dosing recommendations—the same dose range can potentially be applied. Nevertheless a cautious approach to both groups is advisable, following the generally accepted approach of “go low and slow”.

## Data Availability Statement

The datasets generated for this article are available on request to the corresponding author.

## Ethics Statement

The studies involving human participants were reviewed and approved by:

- the Republic of Bulgaria Ministry of Health Ethics Committee for Multicenter Trials,
- the National Medical Research Centre for Psychiatry and Neurology named after V.M. Bekhterev,
- the Ethics Committee of the State Healthcare Institution Saratov City Clinical Hospital #2 named after V.I. Razumovsky,
- the Ethics Committee of the State Budgetary Healthcare Institution of Sverdlovsk region “Sverdlovsk Regional Clinical Psychiatric Hospital”,
- the Ethics Committee of the State Healthcare Institution “Regional Clinical Psychiatric Hospital of St. Sofia”,
- the Ethics Committee of “Scientific-Educational Center for Psychotherapy Podderzhka”.

Written informed consent to participate in this study was provided by the participants' legal guardian/next of kin.

## Author Contributions

BSz, ÁB, JH, IL, BSe, MG, KS and GN have made substantial, direct, and intellectual contribution to the work and approved it for publication.

## Funding

The EudraCT Number: 2016-002327-29 study was sponsored by Gedeon Richter Plc (Budapest, Hungary) and the study NCT01625897 was sponsored by Mitsubishi Tanabe Pharma Corporation (Tokyo, Japan).

## Conflict of Interest

Authors BSz, ÁB, JH, IL, BSe, MG and GN are employed by the company Gedeon Richter Plc. Author KS is employed by the company Mitsubishi Tanabe Pharma Corporation. The authors declare that study RGH-188-201 received funding from Gedeon Richter Plc. and study A002-A7 received funding from Mitsubishi Tanabe Pharma Corporation. The funder had the following involvement in the study: study design, data collection and analysis, decision to publish and the preparation of the manuscript.
